# Crowd‐powered histopathological analysis of brain samples from persons with Alzheimer disease: a Citizen Science pilot study inclusive of Hispanic communities

**DOI:** 10.1002/alz70855_105964

**Published:** 2025-12-24

**Authors:** Pietro Michelucci, Lisa Gusman, Laura Onac, Paz Santander, Margaret Lane, Ravish Dussaruth, Gretė Vaičaitytė, Jerry Jierui Lou, Shino Magaki, Michael Keiser, Brittany N Dugger

**Affiliations:** ^1^ Human Computation Institute, Ithaca, NY, USA; ^2^ University of California, Irvine, Irvine, CA, USA; ^3^ University of California, Los Angeles, Los Angeles, CA, USA; ^4^ University of California San Francisco, San Francisco, CA, USA; ^5^ University of California, Davis, Sacramento, CA, USA

## Abstract

**Background:**

Deriving research‐grade histopathological annotations from multi‐source whole slide images (WSI) entails data quality requirements exceeding today's best automated methods. We seek to address this gap by implementing methods of citizen science, which entails recruiting public volunteers to collaborate in the scientific process, not as human subjects, but as research collaborators. By engaging many volunteers, citizen science provides an opportunity to acquire expert‐like histopathological evaluations in a scalable manner. This approach intrinsically lends itself to community outreach and education. This can especially be impactful in communities disproportionately affected by Alzheimer's disease, such as the Hispanic community. Toward this end, we piloted new human computation methods to engage public volunteers in annotating amyloid pathologies in WSIs of the human brain.

**Method:**

Our pilot study utilized a crowd‐powered analytic pipeline breaking down histopathological analysis into a step‐wise series of manageable online tasks that can be accomplished by non‐expert public volunteers. We also incorporated “wisdom of crowd” methods for each step of the analysis aggregating multiple individual annotations for a single pathological instance into a single expert‐like annotation. This pilot compared our two‐stage crowd‐based analysis to gold standard data generated by neuropathology experts for two pathology categories: cored plaques and cerebral amyloid angiopathy.

**Result:**

Through methodological development, including testing of annotation interfaces and consensus methods, we found with sufficient aggregation of non‐expert answers, it is possible to consistently achieve a level of agreement with gold standard data meeting or exceeding interrater agreement among expert annotators for both stages of analysis

**Conclusion:**

Our approaches support the rapid production of high‐volume annotation datasets for use in machine learning algorithm development to provide deeper phenotyping of neurodegenerative diseases. These pilot results support the plausibility of moving forward with an openly available online platform, which will be used to execute the first ever de‐novo analysis of human brain histopathology images by a crowd of volunteers. This platform will be geared towards impacted populations, such as those of Hispanic heritage, by providing a culturally responsive outreach and inclusion program for such communities to represent themselves in discussions with researchers.

